# The role of living arrangements in disability assistance and survival in Mexican older adults

**DOI:** 10.1093/geroni/igaf147

**Published:** 2025-12-15

**Authors:** Jose Eduardo Cabrero-Castro, Octavio Bramajo, Mariana Calderón-Jaramillo, Philip Cantu, Brian Downer

**Affiliations:** Department of Population Health and Health Disparities, School of Public and Population Health, The University of Texas Medical Branch, Galveston, Texas, United States; Sealy Center on Aging, The University of Texas Medical Branch, Galveston, Texas, United States; Department of Population Health and Health Disparities, School of Public and Population Health, The University of Texas Medical Branch, Galveston, Texas, United States; Sealy Center on Aging, The University of Texas Medical Branch, Galveston, Texas, United States; Center for Demographic Studies, Autonomous University of Barcelona, Barcelona, Spain; Department of Population Health and Health Disparities, School of Public and Population Health, The University of Texas Medical Branch, Galveston, Texas, United States; Sealy Center on Aging, The University of Texas Medical Branch, Galveston, Texas, United States; Department of Population Health and Health Disparities, School of Public and Population Health, The University of Texas Medical Branch, Galveston, Texas, United States; Sealy Center on Aging, The University of Texas Medical Branch, Galveston, Texas, United States

**Keywords:** Life expectancy, Disability (ADL/IADL), Mortality, Mexico

## Abstract

**Background and Objectives:**

This study estimates life expectancy with basic activities of daily living (ADL) and instrumental ADL (IADL) limitations by living arrangements at age 60 for Mexican adults, using the Mexican Health and Aging Study (MHAS) data from 2012 to 2021. We extend previous research by examining assistance with ADL and IADL as a measure of disability severity and exploring the association of disability assistance and mortality.

**Research Design and Methods:**

Generalized estimating equations were used to examine the relationship between receiving help and living arrangements (living alone, with spouse only, or in extended households). Cox proportional hazards regression assessed the effect of receiving help on mortality. Multistate life table models were used to estimate life expectancy with and without help for ADL and IADL limitations, by gender and living arrangements.

**Results:**

At age 60, those living alone spent a larger share of post-disability life without receiving assistance (ADL: 68.1%; IADL: 19.9%) than those living with others (ADL: 61.6%; IADL: 15.8%). Compared with co-residers, older adults living alone had higher odds of not receiving help with ADL and IADL. Individuals receiving help had higher mortality (ADL hazard ratio [HR] = 1.57; IADL HR = 1.62), consistent with help being a marker of greater severity, not a causal effect.

**Discussion and Implications:**

Extended households enable individuals with disabilities to receive assistance for a longer period. Disability assistance was associated with increased mortality risk, highlighting its role as an indirect measure of disability severity.

Innovation and Translational Significance: We quantify unmet care in Mexico’s aging population by partitioning remaining life expectancy at age 60 into three states—healthy, with ADL/IADL limitations and no help, and with limitations and help—using multistate life tables. Individuals living alone spend a larger share of their post-disability life without assistance. In adjusted models, receiving help is associated with higher mortality, indicating greater underlying disability severity rather than harm from assistance. These estimates identify a high-priority group for practice and policy: targeted outreach to those living alone, caregiver support, and expansion of home-based services to reduce unmet need and improve late-life outcomes.

Recent research highlights the profound impact of population aging on health systems: longer life expectancy, rising multimorbidity, and increased pressure on already strained resources (López-Ortega, 2024). In Latin America, demographic aging is proceeding far more rapidly than in most high-income countries (Palloni & Souza, 2013).

In demographic and aging research, care needs are typically operationalized as limitations in activities of daily living (ADLs) and instrumental activities of daily living (IADLs). Although ADLs/IADLs are standardized measures that enable population monitoring and cross-study comparison, families in Mexico routinely perform broader, non-instrumental care tasks; we therefore frame ADLs/IADLs as a measurable subset of a wider caregiving ecosystem (Instituto Nacional de Estadística y Geografía [INEGI] & Instituto Nacional de las Mujeres, 2019; Panel on New Directions in Social Demography et al., 2013).

In nationally representative MHAS data pooled across 2001–2021, about 22% of Mexican adults aged 50+ report at least one ADL limitation, while 16% report at least one IADL limitation (Sáenz et al., 2025). These prevalences imply a sizable population with care needs. In practice, movement from functional limitation to dependence occurs when tasks can no longer be performed independently, generating sustained demand for assistance that must be met either by families or by formal long-term care (LTC) services (Colombo et al., 2011).

In Mexico, where a comprehensive public LTC system has not yet been established, families, especially women, are the main providers of assistance to older adults (Díaz-Venegas et al., 2023). The labor market further shapes caregiving capacity. According to the National Survey of Occupation and Employment (ENOE), informal employment encompasses roughly 54.8% of workers (33 million people), with persistent regional and gender disparities (INEGI, 2025). Informal workers typically lack paid leave and social protection, making it harder to reconcile paid work with sustained caregiving (Samaniego De La Parra & Fernández Bujanda, 2020). In Mexico’s time-use statistics (ENUT, 2019), women devote far more hours to unpaid domestic and care work than men: for care of household members, women report 28.8 hr/week versus 12.9 hr for men; overall unpaid household work creates a 15.9-hr weekly gap (women > men), while men’s market work exceeds women’s by 9.8 hr/week (INEGI & Instituto Nacional de las Mujeres, 2019). Moreover, 50% of the population aged ≥12 provides unpaid care to household members, averaging 9.3 hr/week (INEGI & Instituto Nacional de las Mujeres, 2019). These time-use asymmetries translate into labor-market effects: using MHAS data, Stampini et al. show that having a parent in need of LTC significantly reduces women’s employment and hours, with no comparable effect for men (Stampini et al., 2022).

The predominance of unpaid family care has measurable economic implications. A recent estimate of the monetary costs associated with the absence of a formal LTC system in Mexico puts the annual burden at approximately US $2.43 billion, incorporating lost labor income among unpaid caregivers and related costs (Gutiérrez-Robledo et al., 2022). Public opinion also reflects ambivalence toward institutional care: the 2023 National Care System Survey (ENASIC) found that 50.6% of respondents disagreed with sending older adults to institutions (even part-time), prioritizing “trained staff” and “good treatment” as desired features in any facility (INEGI, 2023). Together, these data emphasize that families are the de facto backbone of care while simultaneously facing constraints that may generate unmet need.

In this context, living arrangements are central. Multigenerational co-residence remains common and is associated with a greater likelihood of receiving support when limitations arise (Díaz-Venegas et al., 2023; González-González et al., 2021). Yet co-residence is not synonymous with sufficiency: constraints on household time and income, especially where members are informally employed, can limit the intensity or continuity of care. International evidence links changes in living arrangements to survival in later life (Feng et al., 2016), and recent multistate life-table work shows how late-life trajectories of independence are structured by household context (Zimmer & Chiu, 2021).

Our analysis leverages these insights within Mexico’s institutional setting, providing the first estimates for Mexico of the life-years older adults spend (1) needing and receiving help, (2) needing but not receiving help, and (3) not needing help, by living arrangement. Our study has three aims. First, we estimate the probability of receiving help with ADLs and IADLs by living arrangement using generalized estimating equations (GEEs). Second, we evaluate the association between living arrangement and mortality using Cox proportional hazards models. Third, we use multistate life-table methods to calculate remaining life expectancy with ADL and IADL limitations, distinguishing between years lived with unmet and met care needs, stratified by living arrangement. By linking functional dependence, help receipt, living arrangements, and survival into a single life-table framework, we provide policy-relevant estimates for a setting where formal LTC is limited, and households absorb the bulk of care.

## Data and methods

### Mexican Health and Aging Study

The Mexican Health and Aging Study (MHAS) is an ongoing, population-based cohort study of aging in Mexico (Wong et al., 2017). The study began in 2001 with a nationally representative sample of 15,186 participants aged 50 and older. A participant’s spouse or partner, regardless of age, was also recruited if they lived in the same household. Follow-up interviews were completed in 2003, 2012, 2015, 2018, and 2021. In 2012, new participants aged 50–59 and their spouses (regardless of age) were added (*n *= 5,896). Additionally, a refreshment sample of participants aged 50–55 and their spouses were added in 2018 (*n *= 4,809). We used data from the 2012 through 2021 observation waves.

We restricted our analytic sample to participants aged 50 or older at the time of the 2012 interview (*n *= 14,923). We further excluded participants lost to follow-up after 2012 (*n *= 613). The final sample sizes for each wave were 14,310 in 2012, 12,915 in 2015, 10,899 in 2018, and 9,719 in 2021, with a total of 10,160 participants (Supplementary Figure 1, see online supplementary material). Of these, 8,810 participants were interviewed in all four waves. Because the three analytic approaches employed required different data structures and inclusion criteria, sample sizes for each analysis are specified in the Statistical Analyses section.

### Measures

#### Limitations in ADLs

The MHAS adapts the Katz Index to assess five basic activities: dressing, bathing, eating, transferring into or out of bed, and using the toilet (Katz et al., 1963). For each activity, respondents indicate whether, because of a health problem, they have any difficulty. We coded each item dichotomously (0 = no difficulty; 1 = difficulty). Respondents who answered “cannot do” or “does not do” were also coded as having difficulty (Cigolle et al., 2007).

We then summed the five items and collapsed the count into three categories: no limitations (0), one limitation (1), and two or more limitations (≥2) following standard MHAS practice (Díaz-Venegas et al., 2016; Garcia et al., 2015).

#### Limitations in instrumental activities of daily living

IADL limitations were measured with four tasks: preparing a hot meal, shopping for groceries, taking medications, and managing money. If respondents reported difficulty or stated they “cannot do” or “does not do” a task, they were asked whether the inability was health‑related. We coded an item as limited when difficulty was reported or a health problem was cited as the reason for non‑performance. The resulting count was classified identically to ADLs: 0, 1, or ≥2 limitations.

#### Receiving help with basic and instrumental activities of daily living

Any respondent who reported difficulty with an ADL or IADL was asked whether someone helps with that task. We defined receiving help as affirmative responses for all tasks in which a limitation was reported. Respondents who lacked help for at least one limited task were classified as not receiving help. We define help receipt as binary, consistent with prior MHAS studies (Díaz-Venegas et al., 2023; Downer et al., 2023). This dichotomy captures whether functional limitations translate into realized care and enables integration into our multistate model of met versus unmet need. Although Garcia et al. (2015) did not analyze help receipt, their active-life-expectancy approach similarly relies on binary state definitions to support life-table estimation. Help variables were constructed separately for ADLs and IADLs.

#### Living arrangements

Household rosters in MHAS record the number of co‑residents, their ages, and their relationship to the respondent. We grouped living arrangements into three categories: (1) Living alone; (2) Living with spouse only; and (3) Extended household—living with at least one other adult (aged ≥15 years) who is a relative or an unrelated adult (Angel et al., 2024).

#### Sociodemographic characteristics

We included age (50–59, 60–69, 70–79, 80–89, ≥90 years), sex, education (0 years, 1–6 years, ≥7 years), marital status (married/civil union, single, divorced/separated, widowed), and housing tenure (owns, rents, borrows).

#### Health characteristics

Self-rated health was grouped into good (excellent/very good/good), fair, and poor. Comorbidity was captured with a dichotomous indicator denoting whether the respondent had ever been told by a health professional that they had *any* of the following: diabetes/high blood sugar, hypertension/high blood pressure, stroke, heart attack, cancer, asthma/emphysema, or arthritis.

### Statistical analyses

1. GEEs: living arrangement and receipt of help

We estimated population-averaged GEEs with a logit link and an exchangeable working correlation, clustering on respondent ID, to test whether living arrangement predicts the likelihood of receiving help among adults who ever reported at least one limitation. Covariates were time-varying and included age group, sex, education, marital status, housing tenure, self-rated health, presence of ≥1 chronic condition, and the number of functional limitations (0, 1, ≥2). Separate models were fit for ADLs and IADLs with Stata’s xtgee.

The analytic sample for the GEE models pooled all person-wave observations across available waves of ADL and IADL data (Supplementary Table 1, see online supplementary material):

- ADL model. Of 8,437 eligible observations, 1,814 lacked self-rated health and were excluded, leaving 6,623 for analysis. Sensitivity models that retained the full sample by omitting self-rated health produced virtually identical estimates.
- IADL model. The initial 6,164 observations were reduced to 5,752 after excluding 412 with missing health status; results were robust to the alternative specification that included all observations.

2. Cox proportional hazards: receipt of help and mortality

Cox proportional hazards models (stcox in Stata) were used to assess the association between receiving help and all-cause mortality over a 9-year period (2012–2021), with “not receiving help” as the reference category. Kaplan–Meier survival curves illustrated unadjusted differences in survival by help status. All models were adjusted for the same set of sociodemographic and health covariates, as well as baseline counts of ADL or IADL limitations. The analytic sample included respondents who reported at least one limitation in ADL or IADL in 2012 (Supplementary Table 1, see online supplementary material).

- ADL cohort: Of 2,202 respondents with ≥1 ADL limitation at baseline, 465 were missing data on self-rated health and were excluded, resulting in 1,737 analytic cases. Findings were unchanged in sensitivity analyses that reintroduced the excluded cases.
- IADL cohort: All 1,448 respondents with baseline IADL limitations had complete data and were included in the analysis.

3. Multistate life-table analysis: life expectancy with and without help

To estimate life expectancy with help and without help/no help (LENH) for ADL and IADL limitations, we used a multistate life table model. This analysis involved a continuous-time Markov process to analyze transitions between states (without limitations, with limitations and receiving help, with limitations and not receiving help, and deceased) at age 60, stratified by sex and living arrangements. Key steps included observing entry into and exit from each state, estimating transition rates, calculating transition probabilities, and predicting state occupation probabilities at the population level. Expected times in each state were computed based on transition rates using methods from the Biography library in R (Willekens, 2014). Our estimates used age 50 as the baseline. The sample size for this analysis included all participants available in each wave: 14,310 in 2012, 12,915 in 2015, 10,899 in 2018, and 9,719 in 2021 (Supplementary Figure 1, see online supplementary material).

## Results

### Sample characteristics

Table 1 shows the baseline characteristics of participants by living arrangements and gender. Women make up a larger proportion of the live alone category (69.3%). Functional limitations are more prevalent among individuals living alone or in extended households than those living with a spouse only. For ADLs, limitations affect 18.8% of women and 14.4% of men living alone, and 18.7% of women and 11.8% of men in extended households, compared to lower rates among those living with a spouse (15.3% for women, 11.4% for men).

**Table 1.** Characteristics of the baseline sample in 2012 by living arrangements and sex.

Variable	Living alone (%)		Living with spouse (%)		Extended household (%)	
	Women	Men	Women	Men	Women	Men
**Number of observations**	943	418	1,467	1,586	5,716	4,180
**%**	69.3	30.7	48.1	57.9	57.8	42.2
**Have ADL limitation(s)**	18.8	14.4	15.3	11.4	18.7	11.8
** Number of limitation(s)**						
** 1**	8.8	6.7	8.7	6.6	9.7	6.1
** 2+**	10	7.7	6.5	4.8	9	5.7
** Have ADL limitation(s) and not receiving help**	75.7	91.7	80.4	80.1	75.1	74.8
**Have IADL limitation(s)**	12.8	8.4	9.8	6.9	13	7.1
** Number of limitation(s)**						
** 1**	7.5	3.6	5.7	4	8	3.7
** 2+**	5.3	4.8	4	2.9	5	3.4
** Have IADL limitation(s) and not receiving help**	34.7	45.7	23.1	26.6	19.7	23.2
**Age**						
** 90+**	2.9	4.8	0.6	1.3	1.5	1.4
** 80–89**	16	15.8	4	8.3	8.2	7.9
** 70–79**	32.4	30.9	20.1	28.5	19	20.4
** 60–69**	30.5	25.8	43.7	43.3	34.3	39.6
** 50–59**	18.2	22.7	31.5	18.6	37	30.7
**Educational attainment**						
** 7+ years**	28.7	32.6	26.9	30.9	26.3	32
** 1–6 years**	47.7	48.4	54.1	51	53.3	52.3
** No formal schooling**	23.7	18.9	19	18.1	20.5	15.7
**Marital status**						
** Married/civil union**	5.5	7.7	98.5	98.7	55.9	82.9
** Single**	12.5	21	0.1	0.1	6.2	2.9
** Divorced/Separated**	20.3	28.7	1.3	1	10.5	4.7
** Widowed**	61.7	42.6	0.1	0.2	27.4	9.6
**Housing tenure**						
** Owns property**	80.9	77.8	86.4	85.2	86.4	87.6
** Rents property**	4.6	6.9	3.1	3.9	3.5	3.3
** Borrowed property**	14.5	15.3	10.5	10.9	10.1	9.1
**Self-rated health status**						
** Excellent, very good, good**	32.6	42.4	34.1	38.7	30.3	42
** Fair**	51.9	46.1	50.8	49.4	55.3	47
** Poor**	15.5	11.5	15.1	11.9	14.4	11
**Prevalence of comorbidities**	68	52.4	67.2	54.5	67.5	52.2

*Note*. ADL = activities of daily living; IADL = instrumental activities of daily living.

Similarly, limitations in IADLs are more prevalent among participants in extended households (13% for women, 7.1% for men) and those living alone (12.8% for women, 8.4% for men) than among individuals living with a spouse (9.8% for women, 6.9% for men). Men living alone face the greatest challenges in accessing support for these limitations, with 91.7% not receiving help for at least one ADL and 45.7% reporting not receiving help for IADL limitations—the highest across all groups.

Age distribution varies by living arrangement. Older adults (80+) are more prevalent among those living alone, with 18.9% of women and 20.6% of men in this age group. Educational attainment tends to be higher among men, with a slightly higher proportion of men achieving more than seven years of schooling across all living arrangements.

Marital status reflects distinct patterns: widowed individuals make up the majority of those living alone (61.7% for women, 42.6% for men) and also represent a substantial group within extended households (27.4% for women, 9.6% for men).

Owning a house is slightly less common among individuals living alone: 80.9% of women and 77.8% of men own a house, compared to ownership rates exceeding 85% among those living with a spouse or in extended households.

Self-rated health and prevalence of comorbidities show consistent trends across groups. Most participants describe their health as fair or good, and comorbidities are more common among women than men, regardless of living arrangement.

### Likelihood of receiving help with ADLs and IADLs

Table 2 presents the results of the GEE linear models estimating the likelihood of receiving care for ADLs and IADLs for participants who reported limitations in these activities (shown in the left and right halves of the table). The odds ratios (OR) from these models indicate the relative risk of receiving help for ADLs and IADLs. ORs below 1 indicate a lower likelihood of not receiving help, while ORs above 1 indicate a higher likelihood of not receiving help. Participants living with their spouses (ADL OR = 0.67, 95% CI: 0.50, 0.91; IADL OR = 0.60, 95% CI: 0.44, 0.80) or in extended households (ADL OR = 0.65, 95% CI: 0.51, 0.82; IADL OR = 0.51, 95% CI: 0.41, 0.63) had lower odds of not receiving help compared to those living alone, for both ADLs and IADLs. This trend is also consistent with single participants, who had higher odds of not receiving help (ADL OR = 1.55, 95% CI: 1.03, 2.32; IADL OR = 1.61, 95% CI: 1.16, 2.23). Women were more likely than men to receive help (IADL OR = 0.85, 95% CI: 0.73, 1.00) but only for IADLs.

**Table 2.** Generalized estimating equations for estimating the likelihood of not receiving help with ADL and IADL, Mexico 2012–2021.

Variable	ADL (*n *= 6,623)				IADL (*n *= 5,752)			
	OR	*p*	**95% CI**		OR	*p*	**95% CI**	
**Living arrangements (Ref: Alone)**								
** Spouse**	0.67	.009**	0.50	0.91	0.60	.001**	0.44	0.80
** Extended Household**	0.65	.000**	0.51	0.82	0.51	.000**	0.41	0.63
**Sex (Ref: Men)**								
** Women**	0.95	.529	0.82	1.11	0.85	.04**	0.73	1.00
**Age (Ref: 90+)**								
** 80–89**	1.77	.000**	1.32	2.38	1.96	.001**	1.31	2.92
** 70–79**	2.50	.000**	1.86	3.36	2.60	.000**	1.74	3.87
** 60–69**	3.03	.000**	2.21	4.14	3.83	.000**	2.54	5.77
** 50–59**	3.32	.000**	2.28	4.83	4.56	.000**	2.92	7.13
**Education (Ref: 7+ years)**								
** 1–6 years**	1.19	.071	0.99	1.43	1.16	.139	0.95	1.42
** No formal schooling**	1.28	.024*	1.03	1.58	1.23	.070	0.98	1.53
**Marital status (Ref: Married/civil union)**								
** Single**	1.55	.034*	1.03	2.32	1.61	.005**	1.16	2.23
** Divorced/separated**	1.16	.242	0.91	1.49	0.96	.736	0.74	1.23
** Widowed**	1.03	.758	0.87	1.22	0.88	.168	0.74	1.06
**Health status (Ref: Excellent, good, very good)**								
** Fair**	1.16	.121	0.96	1.39	1.09	.390	0.90	1.33
** Poor**	1.28	.016*	1.05	1.56	1.13	.281	0.91	1.40
**Comorbidities (Ref: No Comorbidities)**								
** Reporting a comorbidity**	0.87	.126	0.73	1.04	0.77	.003**	0.65	0.91
**Housing tenure (Ref: Owns)**								
** Rents**	0.73	.102	0.50	1.07	0.88	.472	0.61	1.26
** Borrows**	1.15	.194	0.93	1.43	1.09	.403	0.89	1.34
**Number of limitations (Ref: No limitations)**								
** 1 ADL**					0.94	.493	0.79	1.12
** 2+ ADL**	1.22	.007**	1.06	1.41	0.86	.058	0.73	1.01
** 1 IADL**	0.53	.000**	0.44	0.64				
** 2+ IADL**	0.16	.000**	0.13	0.18	1.11	.148	0.96	1.29
**Constant**								
** Intercept**	3.87	.000	2.50	5.99	0.20	.000	0.12	0.33

*Note*. ADL = activities of daily living; IADL = instrumental activities of daily living.

** *p *< .01.

* *p < *.05.

The odds of not receiving help are associated with younger age, for both ADLs and IADLs. Younger participants (aged 50–59) have the highest odds of not receiving help (OR = 3.32, 95% CI: 2.28, 4.83), with older participants having the lowest odds. Participants with ADL limitations and no formal schooling are less likely to receive help (OR = 1.28, 95% CI: 1.03, 1.58), compared to those with seven or more years of schooling but not for IADLs. Older adults with poor self-rated health had higher odds of not receiving help (OR = 1.28, 95% CI: 1.05, 1.56) compared to participants who rated their health as excellent, very good, or good.

Supplementary Figure 2 illustrates the marginal effects on the probability of not receiving help for ADL and IADL limitations by living arrangement. The differences between living arrangement groups indicate the change in the probability of not receiving help when transitioning from one living arrangement to another, while controlling for other variables. Participants with ADLs have a higher probability of not receiving help compared to those with IADLs. Those living alone have the highest probability of not receiving help (ADL: 83%, 95% CI: 80.2, 85.9; IADL: 31%, 95% CI: 27.1, 35.8), whereas those in extended households have the lowest probability (ADL: 77%, 95% CI: 75.9, 78.1; IADL: 19%, 95% CI: 17.9, 20.4). The probability for individuals living with a spouse only is similar to that of those in extended households, with ADL at 78% (95% CI: 75.0, 80.1) and IADL at 22% (95% CI: 18.7, 24.7).

### Mortality risk by receipt of help

Table 3 shows the results of the Cox proportional hazards models estimating the impact of receiving help with ADL and IADL limitations on mortality. Hazard ratios (HRs) greater than 1 indicate an increased risk of death. Receiving help with ADLs was associated with a 57% increase in mortality risk (HR = 1.58, 95% CI: 1.32, 1.88), while receiving help with IADLs was linked to a 62% increase (HR = 1.62, 95% CI: 1.33, 1.97), after adjusting for sociodemographic and health variables. Each additional year of age was associated with a 6% increase in mortality risk in the ADL model (HR = 1.06, 95% CI: 1.05, 1.07) and a 5% increase in the IADL model (HR = 1.05, 95% CI: 1.04, 1.06). Women had a lower mortality risk compared to men, with reductions of 39% in the ADL model (HR = 0.60, 95% CI: 0.51, 0.71) and 42% in the IADL model (HR = 0.57, 95% CI: 0.49, 0.68).

**Table 3.** Estimates of Cox PH model on the odds of dying in Mexico, 2012–2021.

Variable	ADL (*n* = 1,737)				IADL (*n* = 1,447)			
	Exp Coeff	*p*	95% CI		Exp Coeff	*p*	95% CI	
**Age**	1.06	.000**	1.05	1.07	1.05	.000**	1.04	1.06
**Help with ADL/IADL (Ref: Do not receive help)**								
** Receives help**	1.58	.000**	1.32	1.88	1.62	.000**	1.33	1.97
**Sex (Ref: Men)**								
** Women**	0.60	.000**	0.51	0.71	0.57	.000**	0.49	0.68
**Living arrangements (Ref: Alone)**								
** Spouse**	1.05	.750	0.77	1.44	1.09	.601	0.79	1.49
** Extended household**	1.00	.973	0.79	1.27	1.00	.989	0.79	1.27
**Education (Ref: 7+ years)**								
** 1–6 years**	1.03	.808	0.81	1.31	0.98	.859	0.77	1.25
** No formal schooling**	1.07	.615	0.83	1.38	0.94	.666	0.73	1.23
**Marital status (Ref: Married/civil union)**								
** Single**	1.22	.318	0.83	1.79	1.19	.393	0.80	1.78
** Divorced/separated**	0.91	.576	0.64	1.28	1.25	.175	0.91	1.71
** Widowed**	1.13	.252	0.92	1.39	1.12	.276	0.91	1.37
**Health status (Ref: Excellent, good, very good)**								
** Fair**	1.11	.366	0.88	1.40	1.28	.034*	1.02	1.61
** Poor**	1.37	.009**	1.08	1.73	1.49	.001**	1.18	1.89
**Comorbidities (Ref: No Comorbidities)**								
** Reporting a comorbidity**	1.46	.000**	1.19	1.80	1.36	.003**	1.11	1.68
**Housing tenure (Ref: Owns)**								
** Rents**	1.11	.676	0.69	1.77	1.32	.184	0.88	2.00
** Borrows**	1.00	.984	0.79	1.26	1.09	.457	0.87	1.35
**Number of limitations (Ref: No limitations)**								
** 1 ADL**					1.03	.738	0.85	1.26
** 2+ ADL**	1.20	.022*	1.03	1.41	1.18	.074	0.98	1.41
** 1 IADL**	1.27	.018*	1.04	1.55				
** 2+ IADL**	1.81	.000**	1.49	2.19	1.73	.000**	1.48	2.02

*Note*. ADL = activities of daily living; IADL = instrumental activities of daily living.

** *p *< .01.

* *p < *.05.

Living arrangements, education, marital status, and housing tenure were not significantly associated with mortality risk. However, poor self-rated health (ADL HR = 1.37, 95% CI: 1.08, 1.73; IADL HR = 1.49, 95% CI: 1.18, 1.89) and comorbidities (ADL HR = 1.46, 95% CI: 1.19, 1.80; IADL HR = 1.36, 95% CI: 1.11, 1.68) were associated with increased mortality risk.

Participants with two or more ADL limitations had a higher mortality risk in the ADL sample (HR = 1.20, 95% CI: 1.03, 1.41). Participants with two or more IADL limitations had an elevated mortality risk in both the ADL and IADL samples (ADL HR = 1.81,95% CI: 1.49, 2.19; IADL HR = 1.73, 95% CI: 1.48, 2.02).

**Figure 1.**
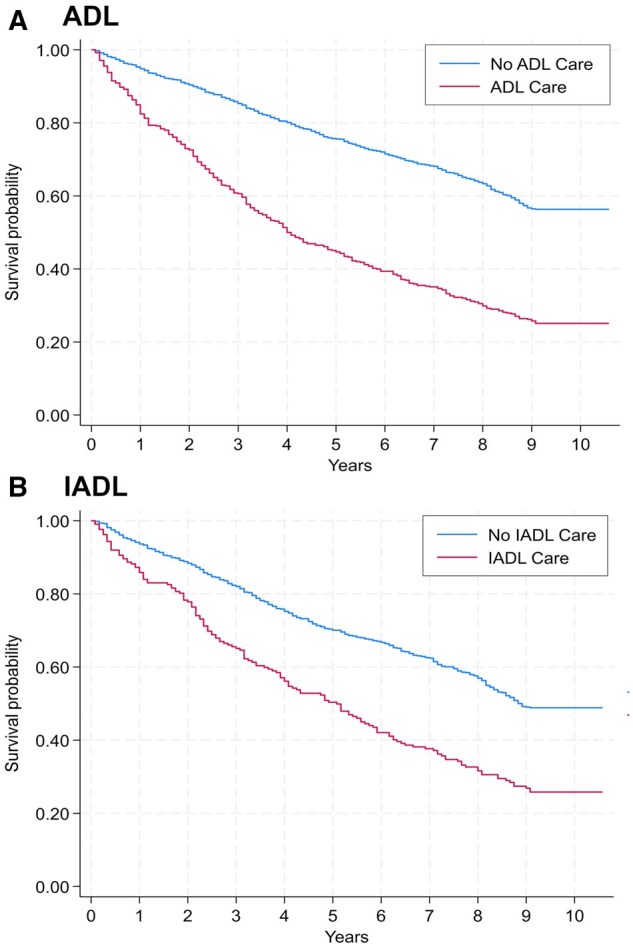
Covariate-adjusted survival curves from a Cox model comparing survival probabilities for those receiving help versus those not receiving help in both ADL and IADL groups. (A) ADL (activities of daily living); (B) IADL (instrumental activities of daily living). Time scale is years since baseline (2012) until death or censoring at last follow-up (2021). Models adjust for age, sex, education, marital status, housing tenure, self-rated health, comorbidity, and number of limitations (0/1/≥2). Interpretation is associative: higher mortality among those receiving care likely reflects greater underlying disability severity, not a causal effect of care.

In general, we found that, depending on having at least one ADL or IADL, receiving help was associated with higher hazards of mortality. Figure 1 illustrates covariate-adjusted survival curves from the Cox model, showing higher mortality among participants who received help with both ADL and IADL tasks compared to those who did not.

### Life expectancy with and without help

Our multistate life tables estimate life expectancy in three states: life expectancy without limitations (healthy life expectancy), life expectancy with limitations but no help received (LENH), and life expectancy with limitations receiving help (LERH). Table 4 presents estimates across these states by living arrangement at age 60. Participants living alone live more years without ADL and IADL limitations compared to those living with a spouse or in extended households (ranging from 1 to 2.6 years).

**Table 4.** Life expectancy at age 60 partitioned into healthy years and years with limitations (by help receipt) by living arrangement, Mexico, 2012–2021.

Sex	Living arrangement	Total LE at 60 (years)	Healthy (HLE)	Limited, No help (LENH)	Limited, Help (LERH)	% of years lived with limitations and receiving help
**Panel A: ADL**						
** Men**	All	25.8	22.4	2.2	1.2	35.3
	Alone	27.5	23.9	2.6	1.0	27.8
	Spouse	27.3	22.9	2.7	1.7	38.6
	Extended	25.2	22.0	2.1	1.1	34.4
** Women**	All	27.4	21.8	3.3	2.3	41.1
	Alone	28.6	23.6	3.2	1.8	36.0
	Spouse	27.2	21.1	3.7	2.4	39.3
	Extended	27.2	21.6	3.3	2.3	41.1
**Panel B: IADL**						
** Men**	All	26.2	23.6	0.5	2.1	80.8
	Alone	28.2	25.6	0.6	2.0	76.9
	Spouse	27.4	24.0	0.5	2.9	85.3
	Extended	25.6	23.1	0.5	2.0	80.0
** Women**	All	28.2	22.9	0.7	4.6	86.8
	Alone	28.9	24.7	0.7	3.5	83.3
	Spouse	27.3	22.1	0.9	4.3	82.7
	Extended	27.8	22.5	0.6	4.7	88.7

*Note*. ADL = activities of daily living; IADL = instrumental activities of daily living. LE values are years remaining at age 60; “Healthy (HLE)” = years without ADL/IADL limitations; “Limited, No help (LENH)” = years with limitations and no assistance; “Limited, Help (LERH)” = years with limitations and assistance. % of years lived with limitations and receiving help = LERH ÷ (LENH + LERH) × 100; percentages refer only to time with limitations, not to total remaining life. Living arrangements: Alone; Spouse (spouse/partner only); Extended (≥1 additional adult).

Participants with ADL limitations live, on average, 1.2 years longer without help than with help, while those with IADL limitations live 2.6 years longer with help than without it. Specifically, participants with ADL limitations spend approximately 64% of their remaining life expectancy without help, whereas those with IADL limitations spend only 17% without help. Women with limitations receive help for a greater proportion of their remaining lives compared to men (ADL: 38.8% vs. 33.6%; IADL: 84.9% vs. 80.7%). Men living with a spouse receive help for 38.6% of their life expectancy with ADL limitations and 85.3% with IADL limitations, which is more than those living alone (ADL: 27.8%; IADL: 76.9%) or in extended households (ADL: 34.4%; IADL: 80%). However, this pattern differs among women. Women living in extended households receive help for 88.7% of their life expectancy with IADL limitations, compared to 83.3% when living alone and 82.7% when living with a spouse only. Finally, participants living alone spend a higher proportion of their time living without help after disability onset. However, for women with IADL limitations, those living with a spouse only had a lower life expectancy receiving help (4.3 years for those living with their spouse and 4.7 years for those living in extended households).

## Discussion

In this study, we examined the need for care of Mexican older adults by estimating the likelihood of receiving help with ADLs and IADLs, the role of living arrangements and receiving help for ADL and IADL limitations on mortality, and the life expectancy of individuals facing ADL and IADL limitations, with or without receiving help. This contribution is particularly salient in Mexico, where long-term care policy remains underdeveloped, fragmented, and heavily reliant on unpaid family caregivers (López-Ortega & Aranco, 2019; Villa, 2019). Unlike many OECD countries, where informal care is complemented by public long-term care systems (Colombo et al., 2011; Vlachantoni, 2019), Mexican families remain the primary, often the only, source of care provision (López-Ortega, 2024). Our study’s key contributions to the literature on care in Mexico include estimates of life expectancy with and without receiving help for ADL and IADL limitations.

Another strength of our study is the use of data from the MHAS, the most comprehensive longitudinal survey on aging in Latin America. While Costa Rica has made efforts with the Costa Rican Longevity and Healthy Aging Study (CRELES); the last follow-up interview took place in 2013. Another country is Brazil with the Estudo Longitudinal de Saúde dos Idosos (Longitudinal Study of the Health of Elderly, ELSI), with only cross-sectional data available (Lee et al., 2021). Our results thus provide evidence from a middle-income country where functional dependence is projected to rise sharply (González-González et al., 2021), but where support remains primarily family-based and public caregiving policies are still emerging (López-Ortega, 2024).

One key finding from our study is how living arrangements affect informal care provision for individuals facing ADL and IADL limitations. Living arrangements are not only associated with the odds of receiving support but also influence life expectancy with receiving help after the onset of limitations. Our results show that individuals who do not live alone—either because they live with a spouse or in an extended household—are more likely to receive help with their limitations in activities (both ADLs and IADLs). In contrast, individuals who live alone tend to spend a greater portion of their remaining lives needing help and not receiving it after their disability onset. These results are consistent with findings from previous studies (Bergh et al., 2023; Spiers et al., 2022) and align with evidence from Mexico showing that dependence is more likely to be met when older adults reside with family members (Díaz-Venegas et al., 2023; González-González et al., 2021). However, this trend may be exacerbated by the reliance on family-provided care in the Mexican context, where multigenerational households remain central to the provision of assistance (Angel et al., 2024; López-Ortega, 2024).

The role of living arrangements in receiving support follows gendered patterns. Our results, consistent with previous studies in the United States (Bergh et al., 2023), Europe (Spiers et al., 2022), and Asia (Momtaz et al., 2012), show that women are more likely to receive help and to receive help for a greater proportion of their lives compared to men. However, women were more likely to receive help when living in extended households, while men were more likely to receive assistance when living with their spouses only, indicating differences in caregiving (and receiving) dynamics. These gendered patterns align with national estimates showing that women perform over 70% of unpaid care hours in Mexico (Villa, 2019), reflecting how the absence of formal caregiver supports exacerbates gender inequalities (Díaz-Venegas et al., 2023). Qualitative studies further underscore the high emotional, financial, and physical burden faced by informal caregivers in Mexico City (Giraldo-Rodríguez et al., 2019), while quantitative research shows that women are more likely to report needing and receiving help compared to men (Díaz-Venegas et al., 2023).

Women likely rely on their adult children or other household members, who are often women themselves, which reflects the sexual division of work and the feminization of care (Herrera & Fernández, 2023). The fact that women tend to receive more help than men can be explained by the gender-survival paradox (Di Lego et al., 2020; Van Oyen et al., 2018): women live longer than men but in poorer health, meaning they spend more time requiring and receiving help than men (Cabrero Castro et al., 2021). Additionally, receiving assistance might indicate the severity of limitations or that women have a higher degree of health-seeking behavior than men (Oksuzyan et al., 2008). However, some of the IADLs in our study, such as the purchase of groceries or meal preparation, may reflect distinct gender expectations in Mexico. It is possible that women are more likely to need assistance because they are more likely to be expected to perform these tasks (Garcia et al., 2015).

While living arrangements were associated with receiving help for ADL and IADL disabilities, they did not have a clear or significant impact on mortality. In our models, living arrangements were not significantly related to mortality but may be associated with it through other aspects like health status. These findings are in line with prior evidence suggesting that survival is shaped more strongly by health conditions and comorbidities than by living arrangements alone (Feng et al., 2016; Freedman et al., 2016). In Mexico, mortality risks among older adults with functional dependence have also been shown to be linked more to socioeconomic status and health burden than to household composition (González-González et al., 2021).

### Limitations

Due to sample size limitations, we were unable to model other aspects relevant to this study, such as transitions between living arrangements or the number of disabilities.

We used care measures that have been used from an absolute approach, which accounts for the availability of support (i.e., whether someone experiencing a limitation is receiving help), and a relative approach, which accounts for the quality of support (i.e., whether the support received is enough to overcome the limitation). The incidence of ADL or IADL limitations, or the development of additional limitations, might result in a change of living arrangement, especially for individuals who live alone or only with their spouses. Self-reports of ADL and IADL limitations, as well comorbidities, are also important data limitations for our study. It is possible that men are less willing to acknowledge their care needs due to cultural standards or because they ignore the need for help with a given activity (Garcia et al., 2015). However, given that we are dealing with strong physical limitations, the likelihood of an unrecognized limitation is rather low, particularly for ADLs.

Additionally, the MHAS does not distinguish if the help provided comes from an informal (family member) or formal (state services or an employee) source. However, given Mexico’s strongly reliance on family structures for caregiving, it is likely that help is provided by of family member or acquaintance, especially for individuals with lower educational attainment and fewer financial resources (López-Ortega & Aranco, 2019). This only further stresses the need for governments and healthcare and welfare institutions to provide reliable and affordable social care services. By estimating the average life expectancy with care needs for daily activities in Mexico—a measure that, to the best of our knowledge, has not been done before—we have not only assessed the intensity of care needs but also the duration for which care is necessary and provided. These findings also acknowledge that such needs may increase in the future, given current population aging trends.

## Conclusion

Mexico’s rapidly aging population and its reliance on family as the main structure for caregiving for older adults exemplify the challenges of meeting growing care needs in the absence of a formal long-term care system. Our study contributes novel evidence by quantifying, for the first time in Mexico, life expectancy with and without help for ADL and IADL limitations, and by examining how these experiences vary by living arrangement. We found that older adults living alone spend a greater proportion of their lives with unmet care needs, while those living with a spouse or in extended households are more likely to receive help. We also showed that receiving help is associated with higher mortality risk, likely reflecting the greater severity of limitations among those receiving assistance.

These findings advance the literature by linking functional limitations, help receipt, and mortality into life-table estimates of met and unmet need. They demonstrate that unmet needs are not evenly distributed but are concentrated among those living alone, while the burden of caregiving falls disproportionately on women and multigenerational households. Translating these findings into practice highlights the urgency of developing caregiver support programs and formal long-term care policies that can complement family caregiving and reduce inequities in access to assistance. By anchoring life-expectancy estimates in the Mexican context, our study provides a baseline for monitoring future reforms and a framework that can be adapted to other low- and middle-income countries facing similar demographic and policy challenges.

Future research should decompose the contributions of economic status to the observed living-arrangement gradients—for example, whether income/wealth and social-protection coverage mediate or modify the probability of receiving help and the duration of unmet need. In parallel, studies should incorporate direct measures of care quality (e.g., adequacy, intensity/continuity of assistance, caregiver training/strain, and formal vs. informal provider type) to distinguish “any help” from *effective* help and to assess how quality differentials shape met and unmet need over time.

## Supplementary Material

igaf147_Supplementary_Data

## Data Availability

Data from the Mexican Health and Aging Study (MHAS) are publicly available upon registration at the following website: https://www.mhasweb.org/Home/index.aspx. This study was not preregistered.
