# Supplementary material for: The role of living arrangements in disability assistance and survival in Mexican older adults
Source: Innov Aging. 2025 Dec 15;10(1):igaf147. doi: 10.1093/geroni/igaf147 (PMC12832939; doi:10.1093/geroni/igaf147)
Supplement: igaf147_Supplementary_Data [file igaf147_supplementary_data.docx]

***Innovation in Aging* Supplementary Material: Cabrero-Castro, Bramajo, Calderón-Jaramillo, Cantu, & Downer. The role of living arrangements in disability assistance and survival in Mexican older adults.**

**Supplementary Table 1.** Distribution of the sample with ADL and IADL limitations across waves.

| **Wave** | **2012** | **2015** | **2018** | **2021** |
| --- | --- | --- | --- | --- |
| Participants | 14,310 | 12,915 | 10,899 | 9,719 |
|  |  |  |  |  |
| ADL | 2,202 | 2,270 | 1,885 | 2,080 |
| IADL | 1,448 | 1,660 | 1,253 | 1,803 |
| Deceased | | 1,021 | 1,072 | 1,538 |
|  |  |  |  |  |
| Receiving help with ADL | 518 | 565 | 743 | 733 |
| Not receiving help with ADL | 1,684 | 1,705 | 1,142 | 1,347 |
|  |  |  |  |  |
| Receiving help with IADL | 1,113 | 1,319 | 1,003 | 1,525 |
| Not receiving help with IADL | 335 | 341 | 250 | 278 |

**Supplementary Figure 1.** Sample selection process for the 2012 baseline wave.

Completed at least one follow-up interview after 2012

N= 14,310

Interviewed in 2015

N= 12,915

Interviewed in 2021

N= 9,719

Deceased between 2018 and 2021 waves

N= 1,538

Lost N= 490

Recovered N= 848

>= 50 years old

N= 14,923

< 50 years old

N= 739

Participants lost to follow-up after 2012

N= 613

Deceased between 2012 and 2015 waves

N= 1,021

Lost N= 374

Interviewed in 2018

N= 10,899

Deceased between 2015 and 2018 waves

N= 1,072

Lost N= 1,296

Recovered N= 352

Participants interviewed in 2012

N= 15,662

Supplementary Figure 2. Average predicted probability of not receiving help with ADL/IADL by living arrangement, based on the GEE model controlling for sociodemographic and health variables.
